# Contribution of socioeconomic, lifestyle, and medical risk factors to disparities in dementia and mortality

**DOI:** 10.1016/j.ssmph.2021.100979

**Published:** 2021-12-09

**Authors:** Jordan Weiss

**Affiliations:** University of California, 2232 Piedmont Avenue, Berkeley, CA, 94720, USA

**Keywords:** Dementia, Intersectionality, Mortality, Multistate, Population attributable fraction

## Abstract

Extensive literature in the United States documents racial/ethnic and gender disparities in the incidence and prevalence of dementia yet few studies have examined how race/ethnicity and gender intersect to shape inequalities in the risk of dementia. Moreover, few studies have examined heterogeneity in the contribution of known risk factors to dementia across these demographic strata while properly accounting for the semi-competing risk of death. I calculated the proportion of dementia cases attributable to socioeconomic, lifestyle, and medical risk factors across demographic subgroups using nationally representative data from the US-based Health and Retirement Study for the years 2000–2016 and a multistate framework that accounts for the semi-competing risk of death. Socioeconomic resources contributed to the largest number of dementia cases but the magnitude of this contribution varied across strata defined by race/ethnicity and gender. The greatest potential for dementia prevention was observed among non-Hispanic black and Hispanic men and women, supporting an intersectionality approach, and underscoring the need for culturally sensitive intervention and public health initiatives to address the growing burden of dementia. Taken together, work demonstrates the potential benefit of taking an intersectional approach to understanding disparities in dementia.

## 1 Introduction

More than an estimated 5.8 million adults aged 65 years and older are living with dementia in 2020 with associated healthcare costs projected to exceed $300 billion (Alzheimer’s Association, 2020). Vital statistics rank dementia as the third most common cause of death in the US (Kramarow & Tejada-Vera, 2019) and it remains the only cause of death in the top 10 without a viable prevention or cure. The burden of dementia is projected to rise in response to population aging (Hebert, Weuve, Scherr, & Evans, 2013; Hurd, Martorell, & Langa, 2015; Matthews et al., 2019) and will be especially pronounced among older adults from diverse racial/ethnic groups who are at increased risk for dementia (Babulal et al., 2019; Haan et al., 2003; Mayeda, Glymour, Quesenberry, & Whitmer, 2016; Rajan, Weuve, Barnes, Wilson, & Evans, 2019) and will make up an increasing share of the US population aged 65 years and older in the coming decades (Colby & Ortman, 2017). In the absence of a cure, and in light of projected demographic changes in the United States population (Colby & Ortman, 2017; Hebert, Scherr, Bienias, Bennett, & Evans, 2003), investigators have turned toward exploring modifiable risk factors that may prevent or delay dementia and disparities therein.

Racial/ethnic disparities in dementia prevalence in the US are well-documented. Studies indicate that the prevalence of dementia may be twice as high among non-Hispanic black (Gurland et al., 1999; Plassman et al., 2007; Potter et al., 2009) and one and one-half times higher among Hispanic (Gurland et al., 1999; Haan et al., 2003; Samper-Ternent et al., 2012) adults relative to age-matched non-Hispanic white adults. Investigators have also reported higher incidence rates of dementia among racial/ethnic minority groups, with non-Hispanic black adults having the highest incidence followed by Hispanic adults and non-Hispanic whites (Mayeda et al., 2016; Shadlen, Larson, Gibbons, McCormick, & Teri, 1999; Yaffe et al., 2013). The mechanisms underlying these disparities remain unclear but are believed to stem from variation in known risk factors for dementia across racial/ethnic groups.

Socioeconomic (e.g., education), lifestyle (e.g., smoking), and medical (e.g., cardiovascular disease) characteristics have all been linked to dementia and dementia-related disparities (Babulal et al., 2019; D. E.; Barnes & Yaffe, 2011; Livingston et al., 2020; Norton, Matthews, Barnes, Yaffe, & Brayne, 2014; Shadlen et al., 1999; Yaffe et al., 2013). Researchers have also investigated whether disparities in dementia may be shaped by genetic pathways but have yet to produce conclusive findings. For example, the primary genetic risk factor for dementia, the APOE-ε4 allele, is more prevalent among blacks than whites but the relative risk of dementia among carriers of the APOE-ε4 allele is lower for blacks than among whites (Marden, Walter, Tchetgen Tchetgen, Kawachi, & Glymour, 2014), further implicating social and cultural characteristics as key determinants of disparities in dementia. Variation in known risk factors may influence disparities in dementia through compositional effects (i.e., prevalence of risk factors), associational effects (i.e., size and strength of the risk factor hazard), or both. Evaluating whether and how these risk factors differentially contribute to dementia across demographic strata could help in shaping strategies to reduce disparities through targeted, culturally sensitive intervention and public health initiatives.

Although a variety of risk factors have been associated with disparities in the onset risk of dementia, little is known about their differential contribution to dementia across racial/ethnic groups. Norton et al. (2014) conducted a cross-national study in which they examined the proportion of dementia cases that could potentially be avoided by reducing exposure to risk factors in socioeconomic, lifestyle, and medical domains, including low educational attainment, smoking, and diabetes. These risk factors were also implicated as key contributors to the burden of dementia in a Lancet report which reviewed evidence from more than 500 scientific peer-reviewed, articles, systematic reviews, and meta-analysis and concluded that 35% of dementia cases may be attributable to modifiable risk factors (Livingston et al., 2020). Though informative, a crucial step to achieving health equity is to understand heterogeneity in the associated magnitude and contribution of these risk factors to dementia across racial/ethnic groups. Efforts to reduce disparities in dementia may also benefit from an intersectional perspective that jointly examines the implications for dementia of race/ethnicity and gender rather than treating these domains as separate or additive categories of analysis.

Many investigators studying racial/ethnic disparities in dementia include gender in their analyses through regression-based adjustment but few have considered race/ethnicity and gender as intersecting categories that shape patterns of disadvantage and social marginalization (e.g., Garcia et al., 2019). The traditional approach of regression-based adjustment tends to ignore the historical, interlocked nature of race/ethnicity and gender by treating these features as separable, additive characteristics thereby positing, for example, that “a woman's racial identity can be ‘subtracted’ from her combined sexual and racial identity” as noted by Spelman (1988). Thus, prior work treating these features as additive may provide a less nuanced understanding of how race/ethnicity and gender interact to shape disparities in the risk of dementia. This may be achieved through an intersectional approach which views the relationship between race/ethnicity and gender as multiplicative (e.g., Weber & Fore, 2007). In the context of intersectionality, for example, non-Hispanic black women would be expected to have poorer health as they are socially disadvantaged along the spectrums of both race/ethnicity and gender (Davis, 1981). Separately examining dementia disparities by race/ethnicity or gender may obscure social pathways through which these disparities emerge. On average, women tend to be at greater risk for dementia compared to men (Podcasy & Epperson, 2016) but this difference is not solely attributable to women's greater longevity (Beam et al., 2018; Michelle M; Mielke, Vemuri, & Rocca, 2014). Although some evidence supports the notion that biological differences contribute to this disparity for some forms of dementia (Altmann, Tian, Henderson, Greicius, & Investigators, 2014; Snyder et al., 2016), it is likely that the higher risk among women is driven by social and cultural dimensions. For example, historically, women have had less access to education than men, and low levels of education are consistently linked to higher risk of dementia for both men and women (Sharp & Gatz, 2011).

Despite their importance, most prior studies of disparities in dementia have focused on race/ethnicity or gender rather than accounting for the possibility that the health effects of race/ethnicity may be contingent on other characteristics, such as gender. An intersectional framework may sharpen our understanding of disparate cognitive health trajectories by contextualizing the multiple levels of stratification that pattern broader health inequalities and access to socioeconomic resources (Williams & Sternthal, 2010). Efforts to reduce disparities in dementia must also account for differential mortality across racial/ethnic and gender groups which may play an important role in explaining the observed disparities; thus, a methodological design that allows for the simultaneous examination of dementia and mortality is warranted.

Modeling associations between incident dementia and its risk factors is complicated by at least two methodological challenges. As is often the case with population-based cohort studies, respondents are interviewed at discrete time intervals; thus, their health status is only observed intermittently (i.e., the data are said to be interval-censored). This inhibits investigators from knowing the exact onset age for the outcome of interest. Conventional survival methods, such as the Cox model, require the age at onset to be known exactly or may otherwise produce biased estimates (Leffondré, Touraine, Helmer, & Joly, 2013). A second methodological challenge is accounting for the semi-competing risk of death. Specifically, when studying longitudinal health-related outcomes among older adults, death is a semi-competing event that may occur prior to (and preclude) or after the non-terminal outcome of interest. Conventional methods for time-to-event data typically assume the absence or independence of competing risks thereby invoking the assumption that, for example, an individual's risk of dementia provides no information about their risk of death. The relative risk of death among persons with dementia compared to those who are dementia free is estimated to be between 1.5 (Jagger, Clarke, & Stone, 1995) and 3.0 (Tschanz et al., 2004) and recent work suggests that life expectancy with dementia may vary by race/ethnicity (Farina, Hayward, Kim, & Crimmins, 2020; Garcia et al., 2019), underscoring the importance of accounting for differential mortality, particularly when studying disparities across demographic strata who themselves exhibit distinct mortality patterns (e.g., Hummer & Gutin, 2018). Moreover, investigators using conventional methods may consider individuals who die prior to developing dementia as censored which violates the assumption of noninformative censoring (Cox, 1972; Kalbfleisch & Prentice, 2011). Incorrect conclusions may be drawn from such analyses as the probability of dementia is estimated—and interpreted—in the absence of mortality which may inflate estimates of the cumulative incidence of dementia (e.g., Berry, Ngo, Samelson, & Kiel, 2010; Lau, Cole, & Gange, 2009) and further obscure social processes underlying disparities in dementia. Taken together, these aforementioned gaps in the literature necessitate an examination of incident dementia through an intersectional lens—which may better reflect the life course patterning of inequalities in dementia and its risk factors—while evaluating how these associations may be altered by the semi-competing risk of death.

The objective of this study was to quantify the contribution of a range of modifiable risk factors to incident dementia across demographic subgroups defined by race/ethnicity and gender while accounting for the semi-competing risk of death. I used observational data from the nationally representative and longitudinal US-based Health and Retirement Study (HRS). Multistate models were used to account for the interval-censored nature of the data and the semi-competing risk of death. I hypothesized that both variation in the prevalence of risk factors across racial/ethnic and gender groups as well as the differential association between risk factors and dementia across subgroups would drive disparities in incident dementia.

## 2 Materials and methods

### 2.1 Study population

The HRS is a nationally representative and longitudinal survey of US adults over the age of 50 and their spouses of any age. Since 1992, the HRS has biennially assessed the economic, health, and social implications of aging through its core survey with response rates greater than 85% in each wave (Sonnega et al., 2014). Baseline interviews were conducted with community dwelling persons but those who transitioned into nursing homes after baseline were retained and interviewed. Black and Hispanic adults were oversampled at about twice the rate of whites, with minority response rates on par with or exceeding those of majority white adults (Mary B Ofstedal & Weir, 2011). Respondents who were unable or unwilling to participate may be surveyed by a proxy respondent (typically a spouse or other family member) who completes the survey on their behalf. Respondent-level sampling weights that account for nursing home residency were calculated and provided by HRS investigators to adjust for the complex sampling design and provide unbiased estimates of population-level parameters (Heeringa & Connor, 1995). The HRS is sponsored by the National Institute on Aging (NIA; U01AG009740) and is conducted by the University of Michigan.

I used nine waves of data from the HRS spanning the years 2000–2016. This time period was selected for its consistent ascertainment of cognitive information. The analytic sample was restricted to adults aged 51 years and older who were dementia-free at baseline in the year 2000 with valid sampling weights and who completed at least one follow-up survey. Respondents who self-reported their race as “Other Race” were excluded due to low sample size.

### 2.2 Outcomes

#### 2.2.1 Dementia ascertainment

The HRS assessed cognitive function with the modified Telephone Interview for Cognitive Status (TICS-M), a global mental status test (Brandt, Spencer, & Folstein, 1988) that can be administered face-to-face or by telephone. The TICS-M was modeled after the Mini-Mental State Exam as described elsewhere (Herzog & Wallace, 1997). The TICS-M includes 10 word immediate (range: 0–10 points) and delayed (range: 0–10 points) recall tests of memory, a serial 7s task (range: 0–5 points) to assess working memory, and a backwards counting from 20 task (range: 0–2 points) to assess attention and processing speed. This results in a composite score which can range from 0 to 27, with higher values indicating better cognitive function. Cut points for dementia were defined using prior HRS studies which were validated against the Aging, Demographics, and Memory Study (ADAMS), a supplemental study of the HRS which involved in-home neuropsychological and clinical assessments combined with expert clinician adjudication to obtain a gold-standard diagnosis of cognitive status (Crimmins, Kim, Langa, & Weir, 2011; Langa et al., 2005). Following the Langa-Weir classification procedure (Crimmins et al., 2011), respondents with scores from 12 to 27 were classified as non-impaired; 7–11 with cognitive impairment without dementia (CIND); and 0–6 with dementia.

Dementia among respondents surveyed by proxy was detected using an 11-point version of the validated Informant Questionnaire on Cognitive Decline in the Elderly (Jorm, 1994, 2004; Jorm, Scott, & Jacomb, 1989; M. B.; Ofstedal, Fisher, & Herzog, 2005). The HRS survey items adapted from the IQCODE included the proxy's assessment of the respondent's memory (excellent [0], very good [1], good [2], fair [3], and poor [4]), ability to perform five instrumental activities of daily living (managing money, taking medication, preparing hot meals, using phones, and shopping for groceries; range: 0–5), and the survey interviewer's assessment of the extent to which the respondent was unable to complete the survey due to cognitive limitations (none [0], some [1], and prevents completion [2]). Proxy respondents with scores of 0–2 were classified as non-impaired; 3–5 with CIND; and 6–11 with dementia (Crimmins et al., 2011).

#### 2.2.2 Mortality ascertainment

Mortality data in the HRS is ascertained through linkages to the National Death Index and from postmortem interviews of a family member of the decedent (typically a spouse, child, or other informant). Current linkage to the National Death Index is available through the end of 2011. In this study, date of death from the National Death Index was used when available or was otherwise obtained from the HRS exit interview. A recent validation study that compared mortality among HRS respondents to US life tables concluded that mortality coverage in the HRS is essentially complete (Weir, 2016).

### 2.3 Risk factors

Risk factors used in the current study were considered to be modifiable, associated with dementia, and known to vary by race/ethnicity or gender. All risk factors were self- or proxy-reported at baseline in the year 2000 and coded *a priori* such that higher scores reflected a higher degree of risk. Missing covariate values were imputed using an iterative, non-parametric technique based on random forests using the missForest package in R (Stekhoven, 2011; Stekhoven & Bühlmann, 2012) which generates a single imputed dataset by averaging over multiple regression trees; thus, it features a multiple imputation framework without the need to run analyses across multiple imputed datasets. This approach has the distinct advantage of accounting for nonlinearity in and interactions between the covariates, and has been shown to outperform commonly used imputation methods including parametric multivariate imputation by chained equations (Stekhoven & Bühlmann, 2012).

#### 2.3.1 Demographic characteristics

Race/ethnicity was determined for each respondent on the basis of their self-report and classified as non-Hispanic white, non-Hispanic black, or Hispanic. Respondents who reported their primary race as “White/Caucasian” and indicated they were not Hispanic were classified as non-Hispanic white. Similarly, respondents who reported their primary race as “Black/African-American” and indicated they were not Hispanic were classified as non-Hispanic black. Adults were classified as Hispanic if they indicated Hispanic origin (Mexican-American/Chicano, Puerto Rican, Cuban American, Other) irrespective of race. There are established sex and gender differences in dementia (Michelle M. Mielke, 2018; Podcasy & Epperson, 2016). With this in mind, and the inclusion of this variable as an indicator that carries both biological and social implications for dementia, I refer to any potential differences between men and women as gender differences (Krieger, 2001). Age was measured in years.

#### 2.3.2 Social and economic resources

Educational attainment was categorized as less than high school or General Educational Development (GED), or otherwise. Occupation was based on the major occupational category of the longest held job reported by the respondent and coded as upper white-collar (i.e., professional, technical, executive), lower white-collar (i.e., sales, administrative support), blue-collar (e.g., operator, service workers, farm), and never worked for pay (Carr, 2012). Respondents reported the safety level of their neighborhood as low (fair or poor) or otherwise (good, very good, or excellent). Respondents also reported whether or not they had enough money to buy the food they needed over the past two years (i.e., food insecurity).

#### 2.3.3 Lifestyle characteristics

Respondents who reported completing vigorous physical activity at least three times per week over the past year prior to their baseline interview were considered physically active as opposed to inactive. Obesity was categorized using body mass index (BMI) and the standard BMI categories developed by the World Health Organization (World Health Organization, 2000) and the National Heart, Lung, and Blood Institute (National Heart, 1998) as underweight (BMI <18.5), normal or overweight (18.5 ≤ BMI <30), or obese (BMI ≥30). Respondents were classified as never smokers, former smokers, or active smokers. US guidelines for alcohol consumption were used to classify respondents as never or low (less than one drink per day for women; up to one drink per day for men) or moderate (one drink per day for women; two drinks per day for men), or heavy (more than eight drinks per week for women; 15+ drinks per week for men) drinkers (U.S. Department of Health and Human Services and U.S. Department of Agriculture, 2015). A single item was used to capture loneliness. Specifically, respondents were asked whether they felt lonely much of the time during the past week prior to their interview.

#### 2.3.4 Medical conditions

Medical conditions were ascertained by asking the respondent whether a medical practitioner had ever informed them of having a chronic condition (e.g., *Has a doctor ever told you that you have diabetes or high blood sugar?*). Medical conditions included diabetes, hypertension, any heart condition (heart attack, coronary heart disease, angina, congestive heart failure, or other heart problems), or stroke. Self-rated hearing was classified as low (fair, poor) or otherwise (good, very good, excellent). Respondent's knowledge of their medical conditions was dependent on their access to and utilization of health services for which there are known disparities across racial/ethnic groups (Nelson, 2002). Thus, additional adjustments were made for whether the respondent had any health insurance (i.e., private insurance, Medicare, or Medicaid) and whether they had visited a doctor or hospital over the past two years.

### 2.4 Statistical analysis

A decision was made *a priori* to stratify all analyses by race/ethnicity and gender based on an intersectional perspective (e.g., Weber & Fore, 2007). In doing so, the models inherently allow for interactions between these demographic strata and all model inputs as well as differential mortality among those with and without dementia. Moreover, there is a large literature documenting disparities in dementia among subgroups defined by these demographic characteristics (e.g., L. L. Barnes & Bennett, 2014; Braveman, 2006; Michelle M. Mielke, 2018; Podcasy & Epperson, 2016; Potter et al., 2009; Wu et al., 2017).

All analyses used the HRS-provided combined person-level and nursing home sampling weights which adjust for the complex survey design and allow estimates to be generalized to the US population of community-dwelling and nursing home adults over the age of 50 (M. Ofstedal, Weir, Kuang-Tsung, & Wagner, 2016; Sonnega et al., 2014). In addition, models were estimated with robust standard errors clustered at the household level to account for the non-independence of observations in the same household. R code and output files are available upon request.

#### 2.4.1 Descriptive analysis

The prevalence of dementia-related risk factors at baseline (survey year 2000) was compared across racial/ethnic and gender groups using Pearson's chi-square test. Non-Hispanic white adults were used as the reference group (e.g., non-Hispanic black men were compared to non-Hispanic white men; Hispanic women were compared to non-Hispanic white women). Gender differences in baseline risk factors were also examined within racial/ethnic groups. Crude incidence rates for dementia were calculated across strata defined by baseline age, race/ethnicity, and gender by dividing the observed number of dementia cases over the study period for each strata by the person-years contributed by each strata. A similar procedure was used to calculate mortality rates among decedents without observed dementia and those with observed dementia over the study period.

#### 2.4.2 Multistate modeling

Population-based studies of incident dementia raise numerous methodological issues. These studies commonly rely on cohorts of older adults who complete cognitive tests at discrete time intervals and for whom the semi-competing risk of death is a concern. In the HRS, for example, progression to dementia is known only to have occurred between two consecutive survey waves; the exact age at dementia onset is not known, and is said to be interval-censored between the diagnostic wave and the previous one (Leffondré et al., 2013). Standard survival analysis techniques, such as the Cox model, do not directly account for interval censoring. Instead, the exact onset age is commonly imputed using the age at the diagnostic wave, the wave prior to dementia onset, or the midpoint between the two which can provide biased estimates (Ahn, Lim, Paik, Sacco, & Elkind, 2018; Law & Brookmeyer, 1992; Pan & Chappell, 2002).

**Fig. 1:**
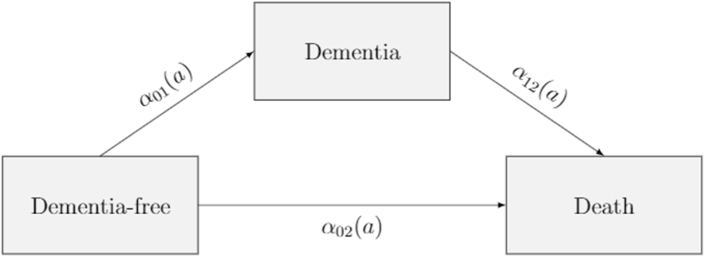
The multistate illness-death model without recovery.

I used a multistate framework to simultaneously evaluate all risk factors (i.e., with full adjustment) while accounting for interval censoring and the semi-competing risk of mortality. Specifically, I modeled a series of parametric irreversible illness-death models for interval-censored data (Joly, Commenges, Helmer, & Letenneur, 2002; Touraine, Helmer, & Joly, 2016). The illness-death model is a three-state model that describes transitions from an initial state (i.e., dementia-free) to an absorbing state (death) either directly or through an intermediate state (dementia). Denote by X=X(a),a≥51 the illness-death process with the state space X(a) at age a defined as 0,1,2 for dementia-free (0), dementia (1), and death (2) and corresponding transitions 0→1,0→2, and 1→2 as illustrated in Fig. 1. The transition intensities $\alpha_{01},\;\alpha_{02},$ and $\alpha_{12}$, which can be interpreted as incidence rates, and regression coefficients were parameterized by a Weibull distribution and estimated simultaneously by maximizing the likelihood function. Hazard ratios were estimated by exponentiating the regression coefficients. Attained age was used as the underlying time scale due to its strong association with incident dementia, with respondent's age at baseline defined as entry time; exit time was defined as age at incident dementia or censoring (i.e., death, or study end). That is, respondents “entered” the study at their baseline age, and “exited” the study either at the age of dementia onset or censoring. Thus, $\alpha_{01}$ corresponds to the age-specific incidence of dementia, $\alpha_{02}$ corresponds to the age-specific mortality rate for adults without observed dementia, and $\alpha_{12}$ corresponds to the age-specific mortality rate for persons following a dementia diagnosis. Importantly, whereas $\alpha_{01}$ and $\alpha_{02}$ feature a respondent's age at baseline as the entry time, the age at dementia onset is used as the entry time for $\alpha_{12}.$

Hazard ratios, standard errors, and confidence intervals were obtained using a clustered bootstrap approach with 1000 replications (Carpenter & Bithell, 2000; Efron, 1992). This provided a distribution for each hazard ratio which was used to estimate the population attributable risk fraction (PAF) and corresponding confidence intervals.

#### 2.4.3 Population attributable risk fractions

Hazard ratios from the multistate model were used to calculate PAFs for each risk factor. Conceptually, the PAF is a counterfactual representation of the fraction by which the occurrence of a condition would be reduced if a given risk factor were eliminated from the population. In this application, the PAF represents the proportion of dementia cases that would be avoided if, for example, all individuals with diabetes at baseline were diabetes-free. Alternatively, the PAF can be interpreted as the percentage of dementia cases attributable to a given exposure level of a risk factor.

PAFs were calculated using a validated method recommended for risk factors with multiple exposure levels (Rockhill, Newman, & Weinberg, 1998):

(1) $$\;PAF=\overset{k}{\underset{i=0}{\sum }}pd_{i}\left(\frac{HR_{i}-1}{HR_{i}}\right)$$

where *i* refers to an exposure level for a given risk factor, *pd* refers to the fraction of dementia cases observed over the study period among individuals in the *i*th risk factor exposure level, and HR refers to the hazard ratio for incident dementia of a given risk factor at a particular exposure level (Rockhill et al., 1998). It is important to note that, albeit counterintuitive, a set of PAFs computed separately for different risk factors are not constrained to sum to one (Rowe, Powell, & Flanders, 2004).

Point estimates and 95% confidence intervals (CIs) for each PAF, for each risk factor, and for each demographic subgroup, were generated using the 1000 bootstrapped HR estimates from the illness-death model (i.e., by estimating 1000 PAFs; taking the mean as the point estimate and taking the values at the 2.5% and 97.5% percentiles as the lower and upper bounds, respectively). Combined domain-specific (i.e., socioeconomic, lifestyle, medical) and overall PAF estimates were also obtained.

PAF estimates and their corresponding 95% CIs were then multiplied by the average annual number of sample-weighted incident dementia cases in the HRS over the study period to calculate the excess number of dementia cases attributable to each risk factor across racial/ethnic and gender groups.

## 3 Results

Among 19,578 individuals who responded to the year 2000 survey wave, I restricted the analytic sample to adults aged 51 years and older at baseline (n = 18,874) with valid sampling weights (n = 18,617) who were dementia-free (n = 17,096). Respondents who self-reported their race as “Other Race” (n = 578) or for whom race or race or Hispanic origin was missing (n = 4) were excluded due to low sample sizes. I further excluded respondents with only one available survey wave (n = 280) resulting in an analytic sample of 16,234 respondents.

### 3.1 Descriptive analysis

Table 1 presents unweighted frequencies and weighted proportions at baseline by race/ethnicity and gender together with results from Pearson's chi-square test which was used to compare the sample-weighted prevalence of risk factors across and within demographic strata at baseline. The analytic sample was 86.4% non-Hispanic white, 8.7% non-Hispanic black, and 4.9% Hispanic. Non-Hispanic white adults had lower levels of socioeconomic disadvantage and fewer lifestyle risk factors whereas medical conditions were mixed across race/ethnicity and gender groups. For example, compared to non-Hispanic white men, non-Hispanic black and Hispanic men reported lower levels of education, were more likely to report food insecurity, be active smokers, and report diabetes but less likely to report a heart condition and poor hearing. Disparities were also observed between men and women within racial/ethnic groups. Non-Hispanic white women, for example, were less likely to report diabetes, a heart condition, and poor hearing relative to non-Hispanic white men. Higher proportions of non-Hispanic black women and Hispanic men reported being high school graduates or higher compared with their race/ethnicity-matched male and female counterparts, respectively, but these proportions were not considered statistically different.

**Table 1:** Descriptive characteristics of the analytic sample at baseline (n = 16,234) by race/ethnicity and gender, HRS 2000.

	NH White		NH Black		Hispanic	
	Men	Women	Men	Women	Men	Women
Characteristic, n (%)	5771 (39.1)	7371 (47.3)	798 (3.6)	1305 (5.1)	424 (2.2)	565 (2.7)
Age						
51–64 y	2488 (52.1)	3098 (45.9)^†^	418 (61.1)*	699 (54.9)*	216 (62.8)*	291 (60.3)*
65–74 y	1914 (27.7)	2260 (28.3)^†^	248 (26.2)*	370 (29.4)*	136 (24.2)*	159 (25.0)*
75–84 y	1111 (17.0)	1561 (20.2)^†^	100 (9.7)*	174 (11.8)*	62 (11.2)*	86 (11.7)*
≥85 y	258 (3.3)	452 (5.6)^†^	32 (3.0)*	62 (3.9)*	10 (1.8)*	29 (3.0)*
*Socioeconomic Resources*						
Educational Level						
Less than high school or GED	1391 (22.0)	1648 (21.2)	385 (46.1)*	564 (42.3)*	268 (58.6)*	380 (62.8)*
High school or above	4380 (78.0)	5723 (78.8)	413 (53.9)*	741 (57.7)*	156 (41.4)*	185 (37.2)*
Occupation						
Never worked for pay	522 (8.9)	1469 (19.3)^†^	223 (27.5)*	484 (38.6)*^†^	135 (32.0)*	339 (57.8)*^†^
Blue-collar	1096 (17.3)	767 (9.8)^†^	196 (22.7)*	195 (13.8)*^†^	144 (28.6)*	82 (14.6)*^†^
Lower white-collar	1037 (18.4)	2271 (30.2)^†^	144 (16.6)*	240 (17.7)*^†^	50 (10.8)*	62 (10.8)*^†^
Upper white-collar	3116 (55.4)	2864 (40.7)^†^	235 (33.2)*	386 (29.9)*^†^	95 (28.6)*	82 (16.7)*^†^
Low neighborhood safety	275 (4.6)	412 (5.2)	180 (22.6)*	312 (23.6)*	65 (14.9)*	97 (16.5)*
Food Insecure	141 (2.8)	274 (4.1)^†^	80 (11.1)*	187 (15.0)*^†^	32 (6.4)*	52 (10.6)*
*Lifestyle Characteristics*						
Physically inactive	2815 (48.3)	4341 (58.6)^†^	462 (57.2)*	848 (66.0)*^†^	226 (50.5)	404 (66.9)*^†^
Body Mass Index						
Underweight	25 (0.4)	231 (3.0)^†^	10 (1.1)*	26 (2.4)*^†^	2 (0.1)	14 (2.3)*^†^
Normal or overweight	4420 (75.5)	5553 (74.9)^†^	555 (70.0)*	729 (53.5)*^†^	309 (72.8)	361 (65.2)*^†^
Obese	1326 (24.1)	1587 (22.1)^†^	233 (28.9)*	550 (44.1)*^†^	113 (26.9)	190 (32.4)*^†^
Smoking						
Never smoked	1557 (28.0)	3695 (49.6)^†^	208 (25.2)*	628 (47.3)^†^	111 (33.1)	352 (60.9)*^†^
Former smoker	3361 (56.3)	2596 (35.0)^†^	407 (48.7)*	471 (35.5)^†^	229 (48.1)	159 (29.8)*^†^
Active smoker	853 (15.7)	1080 (15.5)^†^	183 (26.1)*	206 (17.3)^†^	84 (18.8)	54 (9.3)*^†^
Alcohol Intake						
Low or moderate	5369 (92.8)	6933 (93.9)^†^	756 (93.7)	1269 (97.0)*^†^	385 (88.9)*	555 (97.6)*^†^
Heavy	402 (7.2)	438 (6.1)^†^	42 (6.3)	36 (3.0)*^†^	39 (11.1)*	10 (2.4)*^†^
Lonely	615 (11.1)	1343 (17.9)^†^	122 (15.7)*	319 (23.9)*^†^	59 (15.9)*	177 (32.2)*^†^
Characteristic, n (%)						
*Medical Conditions*						
Diabetes	824 (13.1)	756 (10.2)^b^	186 (22.0)^a^	314 (23.0)^a^	88 (20.4)^a^	111 (16.4)^a^
Hypertension	2518 (42.0)	3229 (42.4)	466 (54.3)^a^	860 (64.7)*^†^	179 (40.1)	268 (42.5)
Stroke	415 (6.4)	471 (6.2)	75 (9.1)^a^	83 (5.9)^b^	22 (5.6)	15 (2.1)*^†^
Heart condition	1568 (24.2)	1341 (17.1)^b^	160 (19.1)^a^	225 (17.3)	55 (13.2)^a^	57 (8.7)^a^
Poor hearing	1608 (25.6)	996 (13.1)^b^	150 (17.2)^a^	176 (13.0)^b^	117 (21.7)	104 (15.0)^b^
*Healthcare Utilization*						
Any insurance	5544 (95.6)	7076 (96.0)	736 (92.5)^a^	1185 (91.6)^a^	352 (77.8)^a^	471 (83.4)^a^
Doctor or hospital visit over past two years	5398 (92.9)	7052 (95.6)^b^	739 (92.2)	1244 (95.5)^b^	377 (88.7)^a^	528 (92.9)^a^

*Notes*: GED, General Educational Development; HRS, Health and Retirement Study; NH, Non-Hispanic.

a *p* < 0.05 for comparison of gender-matched racial/ethnic group to NH whites using Pearson's chi-square test.

b *p* < 0.05 for comparison of gender within racial/ethnic group using Pearson's chi-square test.

Table 2 presents strata-specific crude incidence rates (IRs) for dementia (Panel A), mortality among decedents without observed dementia over the study period (Panel B), and mortality among decedents with observed dementia over the study period (Panel C) calculated using the observed HRS data. Over 198,017 person-years of follow-up (median [interquartile range] length of follow-up, 15.0 [8.0–17.0] years), there were 3349 cases of incident dementia (crude incidence rate: 16.9/1000 person-years). Among the 7906 deaths observed over follow-up, 2179 (27.6%) were among respondents who died with a dementia diagnosis over the study period and 5727 (72.4%) were among decedents without observed dementia over the study period.

**Table 2:** Observed crude incidence rates for dementia (Panel A), mortality among decedents without observed dementia over the study period (Panel B), and mortality among decedents with observed dementia over the study period (Panel C) by race/ethnicity, gender, and baseline age group.

Panel A: Observed crude incidence rates^a^^,^^b^ for dementia by race/ethnicity, gender, and baseline age group.						
	NH White		NH Black		Hispanic	
Baseline age group	No. of Events/No. at Risk	IR (95% CI)	No. of Events/No. at Risk	IR (95% CI)	No. of Events/No. at Risk	IR (95% CI)
51-64 y						
Men	192/2488	4.9 (4.2, 5.8)	95/418	15.7 (12.2, 20.5)	32/216	10.8 (5.9, 21.5)
Women	229/3098	4.5 (3.9, 5.3)	138/699	14.6 (12.0, 17.8)	80/291	16.3 (12.3, 21.8)
65-74 y						
Men	345/1914	16.0 (14.4, 17.8)	98/248	45.9 (37.2, 57.2)	46/136	34.8 (26.2, 47.1)
Women	455/2260	17.1 (15.6, 18.8)	163/370	49.9 (42.8, 58.4)	67/159	42.5 (33.1, 55.1)
75-84 y						
Men	296/1111	34.4 (30.8, 38.5)	41/100	66.4 (49, 90.9)	27/62	55.7 (37.1, 85.3)
Women	584/1561	43.5 (40.3, 47.0)	103/174	83.4 (71.3, 97.7)	49/86	75.3 (55.9, 101.8)
≥85 y						
Men	65/258	47.9 (37.8, 61.2)	11/32	74.0 (38.3, 149.8)	4/10	106.1 (35.6, 346.2)
Women	180/452	79.4 (69.2, 91.4)	32/62	116.8 (85.8, 160.3)	17/29	107.6 (65.6, 177.2)
Panel B: Mortality rates^a,b^ among decedents without observed dementia over the study period by race/ethnicity, gender, and baseline age group.						
	NH White		NH Black		Hispanic	
Baseline age group	No. of Events/No. at Risk	MR (95% CI)	No. of Events/No. at Risk	MR (95% CI)	No. of Events/No. at Risk	MR (95% CI)
51-64 y						
Men	636/2296	17.9 (16.3, 19.5)	116/323	24.5 (19.6, 30.8)	46/184	13.4 (9.4, 19.6)
Women	550/2869	12.3 (11.2, 13.5)	151/561	20.5 (17.0, 24.8)	34/211	10.5 (6.9, 16.5)
65-74 y						
Men	885/1569	48.4 (45.4, 51.5)	100/150	68.2 (54.6, 85.3)	52/90	53.0 (39.9, 71.1)
Women	796/1805	36.4 (34.1, 39.0)	93/207	41.8 (33.4, 52.6)	35/92	31.7 (22.9, 44.7)
75-84 y						
Men	729/815	108.2 (101.9, 114.9)	56/59	145.2 (111.3, 187.1)	30/35	118.5 (85.4, 163.8)
Women	792/977	89.9 (84.7, 95.3)	64/71	112.9 (88.5, 143)	33/37	99.2 (75.8, 128.8)
≥85 y						
Men	192/193	196.7 (177.4, 217.5)	21/21	183.4 (126.1, 257.6)	6/6	191.1 (82.6, 375.8)
Women	268/272	184.0 (169.2, 199.8)	30/30	247.8 (185.5, 322.6)	12/12	155.3 (100.4, 228.5)
Panel C: Mortality rates^a^^,^^b^ among decedents with observed dementia over the study period by race/ethnicity, gender, and baseline age group.						
	NH White		NH Black		Hispanic	
Baseline age group	No. of Events/No. at Risk	MR (95% CI)	No. of Events/No. at Risk	MR (95% CI)	No. of Events/No. at Risk	MR (95% CI)
51-64 y						
Men	84/192	88.7 (68.7, 115.0)	40/95	66.9 (44.9, 100.9)	16/32	125.4 (46.4, 412.6)
Women	91/229	84.6 (67.9, 105.8)	42/138	46.1 (31.9, 67.7)	17/80	33.1 (18.2, 64.3)
65-74 y						
Men	231/345	169.2 (150.1, 190.5)	62/98	130.6 (99.6, 171.4)	27/46	132.7 (89.4, 201.4)
Women	251/455	141.6 (126.2, 158.9)	88/163	95.0 (77.7, 116.6)	24/67	56.0 (36.4, 88.4)
75-84 y						
Men	258/296	230.8 (208.9, 254.7)	36/41	199.2 (158.7, 247.6)	22/27	127.6 (91.5, 177.6)
Women	468/584	207.7 (192.8, 223.7)	86/103	162.2 (139.4, 188.5)	36/49	122.2 (89.5, 165.3)
≥85 y						
Men	64/65	398.0 (312.5, 500.2)	11/11	333.4 (235.1, 468.4)	4/4	203.2 (101.3, 358.5)
Women	177/180	303.7 (274.9, 334.8)	28/32	187.7 (121.6, 281.4)	16/17	208.2 (116.5, 358.4)

*Notes*: MR, Mortality Rate; NH, Non-Hispanic.

a Per 1000 Person-Years.

b Unweighted counts and weighted IRs are shown.

**Fig. 2:**
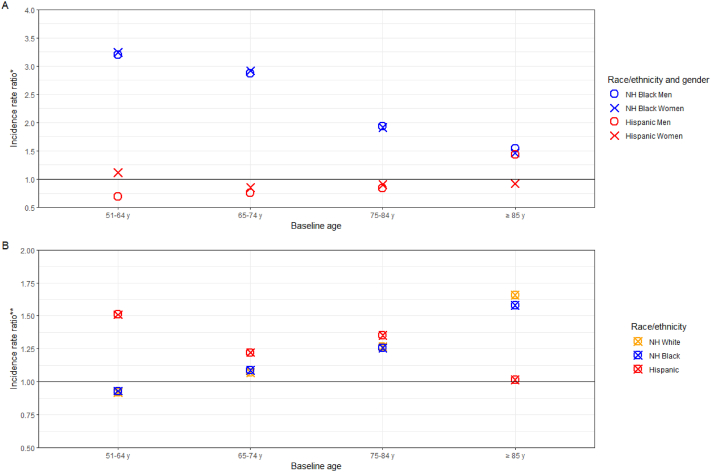
Incidence rate ratios across and within racial/ethnic groups by baseline age. *Notes*: NH, Non-Hispanic. * Panel A depicts the incidence rates for specific subgroups relative to gender-matched non-Hispanic white adults. ** Panel B depicts the incidence rates for men relative to race/ethnicity-matched women.

There were notable differences in the IRs of dementia across racial/ethnic and gender groups at all ages. For example, the incidence rate ratio (IRR) for non-Hispanic black men aged 51–64 years at baseline compared with non-Hispanic white men in the same age group was 3.2, indicating non-Hispanic black men in this age group were 3.2 times more likely than non-Hispanic white men in the same age group to develop dementia over the study period. The IRR for Hispanic men aged 51–64 years at baseline relative to non-Hispanic white men was 2.2. Within racial/ethnic groups and among those aged 75–84 years at baseline, non-Hispanic white and black women were nearly 26% more likely to develop dementia than their male counterparts whereas Hispanic women in this age group were 35% more likely than Hispanic men to develop dementia. The relative risk among non-Hispanic white women aged 85 years and older was nearly 66% higher than non-Hispanic white men; the largest observed gender disparity within racial/ethnic groups. The disparity in the risk of dementia among non-Hispanic black and Hispanic men and women relative to non-Hispanic white men and women declined with advancing age with the exception of Hispanic men aged 85 years and older at baseline whereas the gender disparity (i.e., higher risk among women) within racial/ethnic groups increased with advancing age for non-Hispanic white and black adults, and declined for Hispanic adults (Fig. 2).

Disparities in mortality among decedents without observed dementia over the study period (Table 2, Panel B) and among decedents with observed dementia over the study period (Table 2, Panel C) were observed across all racial/ethnic, gender, and age groups. Among men aged 65–74 years at baseline, the mortality rate (MR) among decedents without observed dementia was highest among non-Hispanic blacks (MR: 68.2; 95%CI: 54.6, 85.3), followed by Hispanics (MR: 53.0; 95%CI: 39.9, 71.1) and non-Hispanic whites (MR: 48.4; 95%CI: 45.4, 51.5). However, among men in the same age group, the mortality rate among decedents with observed dementia was highest among non-Hispanic whites (MR: 169.2; 95%CI: 150.1, 190.5), followed by Hispanics (MR: 132.7; 95%CI: 89.4, 201.4) and non-Hispanic blacks (MR: 130.6; 95%CI: 99.6, 171.4) although the overlapping confidence intervals could indicate these differences were not statistically different. A similar trend was observed for women aged 50–64 years at baseline. That is, among decedent women aged 50–64 years at baseline who did not develop dementia over the study period, non-Hispanic black women had the highest mortality rate followed by Hispanic and non-Hispanic white women. Among decedent women in this age group who developed dementia over the study period, non-Hispanic white women had the highest mortality rate, followed by non-Hispanic black and Hispanic women. The mortality rate ratio for women aged 65–74 years at baseline with observed dementia relative to those without was 3.9 for non-Hispanic white women, 2.3 for non-Hispanic black women, and 1.8 for Hispanic women.

### 3.2 Multistate modeling

Table 3 presents the race/ethnicity and gender specific hazard ratios (HRs) for dementia and corresponding 95% confidence intervals (CIs) for each risk factor obtained from the fully adjusted and stratified multistate models. Risk factors with CIs that contain 1.0 are not considered to be statistically different from one. The overall pattern of results presented in Table 3 demonstrates variation in the magnitude of the associations between risk factors and incident dementia across and within racial/ethnic and gender groups. The hazard for dementia associated with attaining less than a high school diploma or GED had the largest magnitude across all risk factors for non-Hispanic white men (HR = 1.97, 95%CI: 1.59, 2.45) and women (HR = 1.59, 95%CI: 1.36, 1.85) as well as Hispanic women (HR = 3.20, 95%CI: 1.54, 6.61) whereas measures of occupational attainment held the largest associated hazard for dementia among non-Hispanic black men and women. Among Hispanic men, never working for pay and being underweight had the largest magnitudes despite wide confidence intervals which may stem from a smaller sample size. Gender differences in the associated magnitude of risk factors were also observed within racial/ethnic groups. For example, among non-Hispanic white adults, loneliness was associated with a 28% (HR = 1.28, 95%CI: 1.13, 1.44) increased hazard for dementia among women but was not associated with an increased hazard for dementia among men.

**Table 3:** Hazard ratios (95% confidence intervals) obtained from multistate model for dementia by race/ethnicity and gender.

	NH White		NH Black		Hispanic	
	Men	Women	Men	Women	Men	Women
Characteristic						
*Socioeconomic Resources*						
Educational Level						
Less than high school or GED	1.97 (1.59, 2.45)	1.59 (1.36, 1.85)	1.31 (0.78, 2.20)	1.76 (1.19, 2.59)	0.92 (0.47, 1.79)	3.20 (1.54, 6.61)
High school or above	1.00	1.00	1.00	1.00	1.00	1.00
Occupation						
Never worked for pay	1.28 (0.94, 1.74)	1.39 (1.16, 1.66)	3.38 (1.82, 6.26)	1.61 (1.02, 2.55)	4.83 (1.97, 11.85)	3.03 (1.24, 7.38)
Blue-collar	1.32 (1.05, 1.66)	1.13 (0.92, 1.40)	2.66 (1.38, 5.12)	2.01 (1.26, 3.21)	3.06 (1.30, 7.21)	1.61 (0.59, 4.40)
Lower white-collar	1.32 (1.06, 1.64)	1.18 (1.02, 1.38)	1.84 (1.00, 3.39)	1.59 (1.09, 2.33)	3.38 (1.19, 9.63)	2.59 (0.98, 6.88)
Upper white-collar	1.0	1.0	1.0	1.0	1.0	1.0
Low neighborhood safety	1.23 (0.91, 1.66)	1.06 (0.85, 1.34)	0.88 (0.62, 1.23)	1.15 (0.91, 1.44)	1.45 (0.86, 2.46)	0.63 (0.41, 0.96)
Food Insecure	1.43 (0.90, 2.28)	1.60 (1.27, 2.02)	1.45 (0.94, 2.23)	1.30 (0.98, 1.73)	2.22 (1.23, 4.01)	1.19 (0.59, 2.40)
*Lifestyle Characteristics*						
Physically inactive	1.10 (0.95, 1.27)	1.07 (0.95, 1.19)	0.93 (0.67, 1.29)	0.90 (0.72, 1.13)	0.75 (0.43, 1.33)	1.52 (1.02, 2.25)
Body Mass Index						
Underweight	0.78 (0.23, 2.68)	0.79 (0.57, 1.09)	0.41 (0.05, 3.37)	1.69 (0.77, 3.72)	6.28 (2.04, 19.33)	1.66 (0.80, 3.47)
Normal or overweight	1.00	1.00	1.00	1.00	1.00	1.00
Obese	0.94 (0.78, 1.14)	0.95 (0.82, 1.10)	0.89 (0.63, 1.26)	1.13 (0.91, 1.41)	0.50 (0.26, 0.96)	1.12 (0.77, 1.64)
Smoking						
Never smoked	1.00	1.00	1.00	1.00	1.00	1.00
Former smoker	0.98 (0.83, 1.16)	1.03 (0.91, 1.17)	1.62 (1.11, 2.37)	1.02 (0.81, 1.29)	1.56 (0.91, 2.68)	1.33 (0.91, 1.96)
Active smoker	1.34 (1.02, 1.76)	1.32 (1.08, 1.60)	1.81 (1.12, 2.92)	1.19 (0.84, 1.69)	1.26 (0.64, 2.49)	1.63 (0.93, 2.87)
Alcohol Intake						
Low or moderate	1.00	1.00	1.00	1.00	1.00	1.00
Heavy	1.28 (0.95, 1.73)	0.89 (0.68, 1.18)	2.23 (1.15, 4.33)	1.14 (0.53, 2.45)	0.80 (0.34, 1.84)	0.87 (0.40, 1.88)
Lonely	0.99 (0.77, 1.26)	1.28 (1.13, 1.44)	1.27 (0.89, 1.82)	1.38 (1.09, 1.74)	1.21 (0.76, 1.94)	1.15 (0.82, 1.61)
Characteristic						
*Medical Conditions*						
Diabetes	1.52 (1.25, 1.86)	1.44 (1.22, 1.71)	1.37 (0.94, 2.01)	1.26 (1.00, 1.60)	2.62 (1.46, 4.71)	1.48 (0.98, 2.24)
Hypertension	0.97 (0.84, 1.13)	1.08 (0.97, 1.21)	1.17 (0.84, 1.61)	1.09 (0.87, 1.37)	0.80 (0.51, 1.24)	0.88 (0.61, 1.27)
Stroke	1.51 (1.19, 1.92)	1.43 (1.20, 1.71)	1.62 (1.03, 2.55)	0.94 (0.59, 1.51)	2.71 (1.37, 5.38)	2.83 (1.28, 6.27)
Heart condition	1.06 (0.90, 1.25)	0.99 (0.87, 1.14)	0.78 (0.54, 1.13)	1.47 (1.15, 1.89)	1.53 (0.86, 2.71)	0.99 (0.53, 1.82)
Poor hearing	1.13 (0.97, 1.33)	1.07 (0.93, 1.23)	1.21 (0.85, 1.72)	0.93 (0.70, 1.24)	1.16 (0.74, 1.81)	1.12 (0.75, 1.68)
*Healthcare Utilization*						
Any insurance	0.44 (0.29, 0.66)	0.81 (0.54, 1.22)	0.55 (0.34, 0.91)	1.22 (0.76, 1.93)	0.69 (0.37, 1.27)	0.81 (0.48, 1.34)
Doctor or hospital visit over past two years	0.91 (0.66, 1.24)	0.73 (0.57, 0.93)	0.91 (0.48, 1.70)	0.59 (0.37, 0.94)	0.68 (0.35, 1.34)	1.08 (0.54, 2.17)

*Notes*: GED, General Educational Development; NH, Non-Hispanic.

Variation across and within racial/ethnic groups was also observed when examining associations between risk factors and mortality among persons with (Table A1) and without (Table A2) observed dementia over the study period. However, due to relatively small sample sizes, these results should be interpreted with caution. Whereas associations between incident dementia and educational and occupational attainment were observed across nearly all demographic strata, the magnitude of their associations with mortality was comparatively faint. More proximate determinants of mortality, such as lifestyle and medical risk factors, tended to be larger in magnitude.

### 3.3 Population attributable risk fractions

PAFs for incident dementia were calculated using Eq. (1) by combining the HRs presented in Table 3 with the proportion of dementia cases observed over the study period among individuals in a given risk factor exposure level for each demographic subgroup. Table 4 and Fig. A1 present the racial/ethnic and gender specific PAFs as percentages to reflect the percentage of incident dementia cases that could be theoretically prevented through the elimination of the corresponding risk factor. Negative PAF values occur when the HR is less than 1.0; thus, they may be interpreted as protective effects (i.e., the proportional increase in dementia cases that could result from moving the effect of the variable). An estimated 20.6% (95%CI: 15.9%, 25.0%) of dementia cases were attributable to attaining less than a high school degree or GED among non-Hispanic white men compared to 12.6% (95%CI: 9.2%, 15.8%) for non-Hispanic white women. Lower occupational attainment contributed substantially more to incident dementia cases among non-Hispanic black (never worked for pay; PAF = 27.6, 95%CI: 20.1, 34.4) and Hispanic (never worked for pay; PAF = 33.5, 95%CI: 25.1, 40.9) men relative to their non-Hispanic white (never worked for pay; PAF = 3.1, 95%CI: −0.4, 6.4) counterparts. Among the medical risk factors, diabetes was notable for its consistent contribution to dementia cases across all groups, ranging from 4.0% (95%CI: 2.5%, 5.5%) for non-Hispanic women to 21.4% (95%CI: 13.3%, 28.8%) for Hispanic men. The combined PAF estimates for socioeconomic resources were largest for all strata, ranging from 26.5% (95%CI: 19.8%, 32.7%) for non-Hispanic white women to 82.1% (95%CI: 65.2%, 90.8%) for Hispanic women, suggesting that 82.1% of dementia cases may theoretically be preventable by improving educational and occupation attainment for Hispanic women while improving neighborhood safety and eradicating food insecurity. Estimates of the contribution of risk factors to mortality among decedents with and without observed dementia are available in Supplemental Tables A3 and A4, respectively.

**Table 4:** Percentage of incident dementia cases attributable to risk factors by race/ethnicity and gender.

	NH White		NH Black		Hispanic	
	Men	Women	Men	Women	Men	Women
Characteristic						
*Socioeconomic Resources*						
Educational Level						
Less than high school or GED	20.6 (15.9, 25.0)	12.6 (9.2, 15.8)	15.8 (−15.9, 38.9)	26.3 (11.5, 38.5)	−6.8 (−82.6, 37.5)	59.9 (34.2, 75.5)
High school or above	Reference	Reference	Reference	Reference	Reference	Reference
Occupation						
Never worked for pay	3.1 (−0.4, 6.4)	8.7 (4.6, 12.6)	27.6 (20.1, 34.4)	18.0 (3.4, 30.3)	33.5 (25.1, 40.9)	52.8 (22.8, 71.2)
Blue-collar	7.5 (2.0, 12.6)	1.7 (−1.0, 4.3)	21.2 (12.4, 29.2)	11.1 (5.8, 16)	27.4 (15.1, 38.0)	5.0 (−3.5, 12.7)
Lower white-collar	3.7 (1.2, 6.2)	4.6 (0.8, 8.2)	5.2 (1.4, 8.9)	5.5 (1.9, 8.9)	7.1 (3.9, 10.1)	3.0 (1.2, 4.9)
Upper white-collar	Reference	Reference	Reference	Reference	Reference	Reference
Low neighborhood safety	1.4 (−0.4, 3.1)	0.4 (−1, 1.8)	−3.2 (−12.5, 5.3)	3.3 (−2.1, 8.3)	6.2 (−1.3, 13.1)	−9.5 (−21.1, 0.9)
Food Insecure	1.2 (−0.1, 2.4)	2.4 (1.5, 3.3)	4.5 (0.0, 8.7)	4.3 (0.1, 8.4)	7.3 (3.7, 10.8)	1.7 (−4.8, 7.7)
*Lifestyle Characteristics*						
Physically inactive	4.7 (−2.2, 11.1)	3.9 (−3.1, 10.5)	−4.5 (−26.8, 13.9)	−7.2 (−25.4, 8.4)	−20.2 (−76.8, 18.3)	26.8 (3.2, 44.7)
Body Mass Index						
Underweight	−0.1 (−0.6, 0.4)	−0.7 (−1.8, 0.4)	−0.5 (−2.2, 1.2)	1.2 (−0.2, 2.6)	0.6 (0.4, 0.7)	1.3 (−0.2, 2.8)
Normal or overweight	Reference	Reference	Reference	Reference	Reference	Reference
Obese	−1.2 (−5.2, 2.7)	−1.0 (−4.0, 1.8)	−3.2 (−13.9, 6.5)	5.3 (−4.1, 13.8)	−17.6 (−43.0, 3.2)	3.9 (−8.9, 15.3)
Smoking						
Never smoked	Reference	Reference	Reference	Reference	Reference	Reference
Former smoker	−1.1 (−11.8, 8.6)	1.1 (−2.9, 4.9)	20.7 (7.0, 32.3)	0.6 (−7.9, 8.5)	19.6 (−1.8, 36.4)	7.0 (−1.4, 14.6)
Active smoker	3.6 (0.7, 6.5)	2.6 (1.0, 4.2)	12.4 (4.7, 19.5)	2.0 (−1.8, 5.7)	3.6 (−6.2, 12.5)	4.2 (0.4, 7.9)
Alcohol Intake						
Low or moderate	Reference	Reference	Reference	Reference	Reference	Reference
Heavy	1.5 (−0.1, 3.1)	−0.5 (−1.7, 0.7)	5.8 (2.6, 8.9)	0.2 (−1.0, 1.4)	−1.8 (−9.5, 5.3)	−0.1 (−0.8, 0.6)
Lonely	−0.2 (−3.4, 2.9)	5.3 (2.9, 7.7)	4.9 (−1.8, 11.2)	7.8 (2.8, 12.5)	4.1 (−5.3, 12.6)	5.3 (−7.2, 16.3)
Characteristic						
*Medical Conditions*						
Diabetes	6.1 (3.7, 8.4)	4.0 (2.5, 5.5)	5.6 (−0.3, 11.2)	5.2 (0.4, 9.8)	21.4 (13.3, 28.8)	8.4 (0.9, 15.3)
Hypertension	−1.2 (−8.1, 5.3)	3.8 (−1.7, 9.0)	8.0 (−8.9, 22.3)	5.7 (−9.9, 19.1)	−11.7 (−40.1, 11.0)	−7.1 (−30.6, 12.2)
Stroke	3.6 (1.9, 5.3)	3.1 (1.8, 4.4)	4.6 (1.2, 7.9)	−0.4 (−3.6, 2.7)	9.4 (5.6, 13.1)	2.9 (1.6, 4.1)
Heart condition	1.8 (−3.0, 6.3)	−0.1 (−3.1, 2.8)	−5.1 (−14, 3.1)	6.9 (3.2, 10.5)	7.4 (−0.9, 15.1)	−0.1 (−5.1, 4.7)
Poor hearing	4.3 (−1.0, 9.3)	1.3 (−1.3, 3.8)	4.0 (−3.0, 10.6)	−1.2 (−6.6, 3.9)	4.0 (−8.2, 14.9)	2.6 (−6.2, 10.7)
*Domain, Combined*						
Socioeconomic Resources	31.9 (27.1, 36.4)	26.5 (19.8, 32.7)	59.8 (47, 69.5)	53.4 (43.3, 61.8)	70.1 (47.4, 83)	82.1 (65.2, 90.8)
Lifestyle Characteristics	7.1 (−7.2, 19.4)	10.4 (2.1, 18.1)	34.6 (10.1, 52.4)	10.2 (−9.1, 26.2)	−4.1 (−79.5, 39.6)	41.5 (18.2, 58.2)
Medical Conditions	13.8 (5.5, 21.4)	11.4 (5.8, 16.7)	16.3 (1.4, 28.9)	15.3 (1.1, 27.4)	27.2 (7.6, 42.6)	7.1 (−13.1, 23.6)
**Total**	45.4 (35.5, 53.8)	41.0 (33, 47.9)	78.7 (67.2, 86.2)	64.1 (51.9, 73.2)	78.2 (57.0, 89.0)	90.3 (79.7, 95.4)

*Notes*: GED, General Educational Development; NH, Non-Hispanic.

Race/ethnicity and gender specific PAFs for incident dementia were applied to the sample-weighted average annual number of dementia cases over the study period (2000–2016) to estimate the number of dementia cases attributable to each risk factor domain for each racial/ethnic and gender group (Table A5). These results suggest that, for example, improving the socioeconomic position of adults could reduce the average annual number of incident dementia cases by 84,126 (95%CI: 62,738, 10,3719) for non-Hispanic white women, 33,654

(95%CI: 62,738, 10,3719) for non-Hispanic black women, and 25,862 (95%CI: 20,538, 28,595) for non-Hispanic women.

## 4 Discussion

Racial/ethnic and gender disparities in dementia and its risk factors are consistently reported across the literature but the extent to which these domains of social inequality intersect to shape the risk of dementia remains understudied, especially in the context of accounting for the semi-competing risk of mortality. Moreover, although biological and genetic markers play an important role in dementia etiology, the emergence of disparities is more likely attributable to the accumulation of socioenvironmental, economic, and health-related experiences that vary across racial/ethnic and gender groups (McDonough & Allen, 2019), underscoring the importance of investigating modifiable characteristics that span these domains. In fact, prior work which reviewed more than 500 scientific peer-reviewed articles, systematic reviews, and meta-analyses estimated that modifiable risk factors accounted for 35% of dementia cases (Livingston et al., 2020). As informative as this work has been, it was not intended to address the matter of disparities in dementia, and the bulk of the studies reviewed were conducted using samples primarily of European ancestry. In the absence of a cure and in light of shifting demographics towards an older and more racially/ethnically diverse population, understanding how these risk factor associations vary across and contribute to racial/ethnic and gender disparities in dementia is increasingly important. One approach to addressing disparities in dementia and the increasingly disproportionate burden of dementia shouldered by non-Hispanic black and Hispanic men and women is to identify intervention targets specific to these demographic strata. Intersectionality provides a framework for understanding the interlocked nature of these subgroups and how they result in unique social contexts and health pathways that emerge over the life course (Holman & Walker, 2020).

In this study, I took an intersectional approach to evaluate racial/ethnic and gender differences in the contribution of modifiable risk factors to incident dementia in a nationally representative sample with up to 16 years of follow-up while accounting for the semi-competing risk of mortality. In doing so, this study offers insight into modifiable pathways underlying racial/ethnic and gender disparities in dementia. Disparities in dementia incidence across subgroups were driven by both variation in the prevalence of risk factors across racial/ethnic and gender groups as well as differences in the associated magnitude between risk factors and dementia incidence across subgroups. For example, 32.2% of Hispanic women reported loneliness compared to 15.9% among Hispanic men yet the magnitude of association between loneliness and incident dementia was stronger for Hispanic men than for women; however, the overlapping confidence intervals suggests that the difference in magnitude was not statistically significant. Among non-Hispanic white adults, the proportion of men and women who reported being high school graduates was similar but the association between education and incident dementia was stronger for men than women.

Across strata defined by race/ethnicity and gender, socioeconomic resources contributed to the largest number of dementia cases compared with the contribution of lifestyle characteristics and medical conditions although there was variation in the magnitude of this contribution. The contribution of lifestyle characteristics and medical conditions to dementia also varied widely across strata. When taking into account socioeconomic resources, lifestyle characteristics, and medical conditions, the total percentage of dementia cases that could theoretically be preventable through modifying risk factors across these three domains was largest for non-Hispanic black and Hispanic men and women. This suggests that the largest potential reduction in the burden of dementia may be achieved through improving risk factors among racial/ethnic underrepresented men and women. These results were consistent with prior work by Barnes and Yaffe (2011) in which the authors calculated attributable risks for dementia of select risk factors using relative risk estimates obtained from meta-analyses combined with population prevalence estimates of specific risk factors including low education, smoking, obesity and hypertension at midlife, and diabetes.

I further quantified the contribution of these risk factor domains to mortality among decedents with and without observed dementia over the study period. I found that lifestyle characteristics contributed to the majority of deaths among non-Hispanic white and Hispanic men and women who did not develop dementia over the study period whereas medical conditions made the largest contribution among non-Hispanic black men and women. Among decedents with observed dementia, the contribution of specific risk factor domains to mortality varied across and within racial/ethnic and gender groups.

Strengths of this study include the use of a large, nationally representative sample of US adults with up to 16 years of follow-up, ascertainment of dementia status using validated criteria (Crimmins et al., 2011; Langa et al., 2005), and mortality coverage that is essentially complete (Weir, 2016). Moreover, the HRS implements a survey strategy that oversamples black and Hispanic adults to promote sample diversity (Sonnega et al., 2014). Second, I used a multistate framework to account for the semi-competing risk of death which has been overlooked in most prior studies of incident dementia. Third, whereas prior work has tended to treat race/ethnicity and gender as additive in nature, I took an intersectional perspective which may better reflect how social statuses intersect to shape disparities in dementia over the life course among non-Hispanic white, non-Hispanic black, and Hispanic men and women.

This study also has several limitations. I calculated the contribution of each risk factor to incident dementia using PAF-based estimates which rely on the assumption of a causal relationship between each risk factor and incident dementia. Despite this assumption, estimates reported in this study should be interpreted with caution and in a limited sense of statistical association. Moreover, the PAF itself is a hypothetical construct that reflects a counterfactual scenario in which a given risk factor is eliminated from a given population (Rockhill et al., 1998). Although complete elimination of a risk factor is unlikely, reduction in risk factors is likely to delay or offset the risk of dementia which could translate to large population-level changes in the prevalence of dementia as reported herein. This study used self- or proxy-reports of medical conditions on the basis of whether the respondent had ever been informed by a physician that they had a given condition. Thus, the results may be affected by response or recall bias. However, prior studies (Jiang et al., 2015; Tisnado et al., 2006) have reported high concordance among self-reported and actual medical conditions which dampens this concern. The exact timing of the dementia diagnosis is unknown as respondent's cognitive status is only observed intermittently. However, the statistical approach used in this study is better able to address the interval-censored nature of the data relative to the more traditional Cox model which requires the age at onset to be known exactly. In addition, concerns that moderate or severe cognitive impairment could affect self-report are mitigated due to the exclusion of participants with dementia at baseline.

An older population age structure in the United States is expected to correspond with an increase in the number of adults with dementia. In recent work, however, scholars have reported a decline in the incidence and prevalence of dementia, attributing these declines to population-level improvements in education and management of cardiometabolic conditions. Results from the current study highlight a greater contribution of these risk factors to dementia across racial/ethnic minority adults which may reflect greater potential for dementia prevention among these groups—especially considering projected increases in the proportion of racial/ethnic minority adults aged 65 years in the coming decades. The potential for greater reduction of dementia among racial/ethnic underrepresented men and women at the population level may reflect the historical marginalization of these subgroups within society that has limited their access to resources through structural barriers underscoring the need for culturally sensitive intervention and public health initiatives that are specific to the context in which they are being implemented. Moreover, these results suggest the need to move from population-level models of dementia to models that are specific to demographic strata which may be more informative for clinicians and policymakers as they grapple with the rising burden of dementia. Findings from this work suggest that socioeconomic resources and healthy lifestyle engagement may not confer the same protection against dementia across racial/ethnic and gender groups. These results could guide public health planning and inform culturally sensitive priorities to reduce disparities in dementia risk in accordance with the National Alzheimer's Project Act (Babulal et al., 2019; US Department of Health and Human Services, 2018). Future analyses using samples with greater racial/ethnic and gender diversity are required to identify and prioritize intervention targets and health guidelines for vulnerable populations more broadly.

## Ethical statement

This research used publicly available, deidentified data. Thus, it was exempt from IRB review.

## Author statement

Jordan Weiss: Conducted all aspects of the study and maintains responsibility for the manuscript.

## Declaration of competing interest

The author declares no conflicts of interest, financial or otherwise.
