# Supplementary material for: Contribution of socioeconomic, lifestyle, and medical risk factors to disparities in dementia and mortality
Source: SSM Popul Health. 2021 Dec 9;16:100979. doi: 10.1016/j.ssmph.2021.100979 (PMC8683757; doi:10.1016/j.ssmph.2021.100979)
Supplement: Multimedia component 1 [file mmc1.docx]

**“Contribution of socioeconomic, lifestyle, and medical risk factors to disparities in dementia and mortality”**

**(Population Health Manuscript Number: SSMPH-D-21-00525)**

**Supplementary Material**

Table A1. Hazard ratios obtained from multistate model for mortality among decedents without observed dementia over the study period by race/ethnicity and gender.

|  |  | NH White | |  | NH Black | |  | Hispanic | |
| --- | --- | --- | --- | --- | --- | --- | --- | --- | --- |
|  |  | Men | Women |  | Men | Women |  | Men | Women |
| Characteristic |  |  |  |  |  |  |  |  |  |
| *Socioeconomic Resources* |  |  |  |  |  |  |  |  |  |
| Educational Level |  |  |  |  |  |  |  |  |  |
| Less than high school or GED |  | 1.12 (0.97, 1.28) | 1.23 (1.08, 1.41) |  | 1.32 (0.72, 2.44) | 1.06 (0.69, 1.63) |  | 0.46 (0.22, 0.97) | 0.87 (0.45, 1.69) |
| High school or above |  | 1.00 | 1.00 |  | 1.00 | 1.00 |  | 1.00 | 1.00 |
| Occupation |  |  |  |  |  |  |  |  |  |
| Never worked for pay |  | 1.20 (0.98, 1.47) | 1.08 (0.94, 1.24) |  | 1.51 (0.77, 2.98) | 1.31 (0.78, 2.21) |  | 1.75 (0.76, 4.04) | 1.27 (0.58, 2.78) |
| Blue-collar |  | 1.29 (1.12, 1.48) | 1.03 (0.87, 1.23) |  | 1.31 (0.69, 2.47) | 1.58 (0.94, 2.68) |  | 1.50 (0.67, 3.39) | 0.88 (0.36, 2.16) |
| Lower white-collar |  | 1.25 (1.10, 1.41) | 1.14 (1.02, 1.26) |  | 1.61 (1.04, 2.49) | 1.11 (0.75, 1.63) |  | 1.01 (0.46, 2.22) | 0.77 (0.33, 1.79) |
| Upper white-collar |  | 1.00 | 1.00 |  | 1.00 | 1.00 |  | 1.00 | 1.00 |
| Low neighborhood safety |  | 0.98 (0.78, 1.24) | 1.05 (0.89, 1.24) |  | 1.47 (1.06, 2.04) | 1.23 (0.94, 1.60) |  | 1.86 (1.05, 3.30) | 0.61 (0.32, 1.18) |
| Food Insecure |  | 1.19 (0.90, 1.56) | 1.42 (1.16, 1.74) |  | 1.43 (0.92, 2.23) | 1.80 (1.31, 2.47) |  | 0.47 (0.14, 1.57) | 2.01 (0.72, 5.56) |
| *Lifestyle Characteristics* |  |  |  |  |  |  |  |  |  |
| Physically inactive |  | 1.44 (1.31, 1.57) | 1.28 (1.17, 1.41) |  | 1.22 (0.91, 1.64) | 1.36 (1.01, 1.84) |  | 1.05 (0.64, 1.70) | 1.39 (0.82, 2.38) |
| Body Mass Index |  |  |  |  |  |  |  |  |  |
| Underweight |  | 1.97 (1.19, 3.29) | 1.75 (1.46, 2.08) |  | 1.4 (0.72, 2.71) | 3.32 (1.96, 5.63) |  | 1.60 (0.51, 4.97) | 3.74 (1.28, 10.9) |
| Normal or overweight |  | 1.00 | 1.00 |  | 1.00 | 1.00 |  | 1.00 | 1.00 |
| Obese |  | 1.15 (1.02, 1.28) | 1.05 (0.94, 1.17) |  | 0.84 (0.59, 1.20) | 1.27 (0.97, 1.65) |  | 1.52 (0.97, 2.39) | 1.25 (0.75, 2.07) |
| Smoking |  |  |  |  |  |  |  |  |  |
| Never smoked |  | 1.00 | 1.00 |  | 1.00 | 1.00 |  | 1.00 | 1.00 |
| Former smoker |  | 1.38 (1.24, 1.53) | 1.37 (1.25, 1.50) |  | 1.32 (0.91, 1.90) | 1.25 (0.94, 1.68) |  | 1.34 (0.79, 2.26) | 1.60 (0.96, 2.67) |
| Active smoker |  | 2.46 (2.11, 2.87) | 2.99 (2.63, 3.41) |  | 2.24 (1.51, 3.33) | 1.85 (1.31, 2.61) |  | 2.67 (1.37, 5.20) | 2.43 (1.23, 4.82) |
| Alcohol Intake |  |  |  |  |  |  |  |  |  |
| Low or moderate |  | 1.00 | 1.00 |  | 1.00 | 1.00 |  | 1.00 | 1.00 |
| Heavy |  | 1.29 (1.08, 1.55) | 1.06 (0.89, 1.26) |  | 0.97 (0.47, 2.03) | 2.33 (1.14, 4.79) |  | 0.82 (0.33, 2.01) | 1.00 (0.20, 5.09) |
| Lonely |  | 1.24 (1.08, 1.42) | 1.26 (1.14, 1.39) |  | 0.84 (0.53, 1.32) | 1.00 (0.77, 1.30) |  | 1.71 (1.02, 2.86) | 0.81 (0.43, 1.50) |

Table A1 (Continued). Hazard ratios obtained from multistate model for mortality among decedents without observed dementia over the study period by race/ethnicity and gender.

|  |  | NH White | |  | NH Black | |  | Hispanic | |
| --- | --- | --- | --- | --- | --- | --- | --- | --- | --- |
|  |  | Men | Women |  | Men | Women |  | Men | Women |
| Characteristic |  |  |  |  |  |  |  |  |  |
| *Medical Conditions* |  |  |  |  |  |  |  |  |  |
| Diabetes |  | 1.53 (1.37, 1.71) | 2.01 (1.77, 2.28) |  | 1.46 (1.05, 2.04) | 2.48 (1.89, 3.27) |  | 4.12 (2.59, 6.56) | 2.56 (1.51, 4.34) |
| Hypertension |  | 1.14 (1.04, 1.25) | 1.31 (1.20, 1.42) |  | 1.25 (0.91, 1.71) | 1.23 (0.92, 1.63) |  | 0.95 (0.61, 1.48) | 0.65 (0.41, 1.02) |
| Stroke |  | 1.49 (1.28, 1.74) | 1.55 (1.34, 1.78) |  | 1.57 (1.02, 2.41) | 1.73 (1.15, 2.62) |  | 1.79 (0.92, 3.49) | 2.61 (0.77, 8.79) |
| Heart condition |  | 1.49 (1.35, 1.64) | 1.50 (1.36, 1.65) |  | 1.97 (1.46, 2.67) | 2.25 (1.67, 3.02) |  | 1.37 (0.80, 2.34) | 2.10 (1.20, 3.69) |
| Poor hearing |  | 0.90 (0.81, 0.99) | 1.10 (0.98, 1.22) |  | 1.02 (0.72, 1.45) | 0.84 (0.60, 1.19) |  | 1.24 (0.75, 2.05) | 1.01 (0.61, 1.66) |
| *Healthcare Utilization* |  |  |  |  |  |  |  |  |  |
| Any insurance |  | 0.71 (0.54, 0.93) | 0.95 (0.71, 1.26) |  | 0.74 (0.43, 1.28) | 0.87 (0.52, 1.46) |  | 0.72 (0.35, 1.45) | 1.34 (0.60, 2.98) |
| Doctor or hospital visit over past two years |  | 0.92 (0.75, 1.12) | 0.81 (0.64, 1.02) |  | 0.90 (0.47, 1.72) | 0.55 (0.31, 0.98) |  | 0.80 (0.36, 1.79) | 1.43 (0.44, 4.71) |

Table A2. Hazard ratios obtained from multistate model for mortality among decedents with observed dementia over the study period by race/ethnicity and gender.

|  |  | NH White | |  | NH Black | |  | Hispanic | |
| --- | --- | --- | --- | --- | --- | --- | --- | --- | --- |
|  |  | Men | Women |  | Men | Women |  | Men | Women |
| Characteristic |  |  |  |  |  |  |  |  |  |
| *Socioeconomic Resources* |  |  |  |  |  |  |  |  |  |
| Educational Level |  |  |  |  |  |  |  |  |  |
| Less than high school or GED |  | 0.85 (0.69, 1.05) | 0.96 (0.84, 1.11) |  | 0.74 (0.42, 1.30) | 0.69 (0.47, 1.02) |  | 0.61 (0.25, 1.47) | 0.93 (0.38, 2.29) |
| High school or above |  | 1.00 | 1.00 |  | 1.00 | 1.00 |  | 1.00 | 1.00 |
| Occupation |  |  |  |  |  |  |  |  |  |
| Never worked for pay |  | 0.69 (0.51, 0.92) | 0.82 (0.69, 0.97) |  | 0.79 (0.38, 1.65) | 0.86 (0.54, 1.37) |  | 1.59 (0.38, 6.59) | 0.44 (0.13, 1.47) |
| Blue-collar |  | 0.75 (0.60, 0.94) | 0.90 (0.74, 1.10) |  | 1.05 (0.45, 2.48) | 0.97 (0.64, 1.48) |  | 0.75 (0.21, 2.64) | 0.15 (0.04, 0.59) |
| Lower white-collar |  | 0.79 (0.64, 0.99) | 0.92 (0.79, 1.08) |  | 0.76 (0.31, 1.85) | 0.77 (0.48, 1.24) |  | 4.06 (1.18, 13.91) | 0.80 (0.24, 2.66) |
| Upper white-collar |  | 1.00 | 1.00 |  | 1.00 | 1.00 |  | 1.00 | 1.00 |
| Low neighborhood safety |  | 1.09 (0.85, 1.41) | 1.01 (0.79, 1.27) |  | 0.78 (0.50, 1.21) | 0.99 (0.74, 1.31) |  | 0.58 (0.32, 1.07) | 0.52 (0.26, 1.05) |
| Food Insecure |  | 0.89 (0.60, 1.32) | 1.16 (0.92, 1.47) |  | 1.19 (0.70, 2.02) | 1.25 (0.90, 1.74) |  | 0.68 (0.30, 1.50) | 1.40 (0.62, 3.16) |
| *Lifestyle Characteristics* |  |  |  |  |  |  |  |  |  |
| Physically inactive |  | 1.16 (1.00, 1.35) | 1.01 (0.90, 1.14) |  | 1.16 (0.80, 1.67) | 1.35 (1.01, 1.81) |  | 1.40 (0.78, 2.51) | 0.64 (0.36, 1.12) |
| Body Mass Index |  |  |  |  |  |  |  |  |  |
| Underweight |  | 2.31 (0.42, 12.58) | 0.88 (0.59, 1.30) |  | 29.6 (5.85, 149.83) | 1.47 (0.70, 3.12) |  | 0.00 (0.00, 0.00) | 2.29 (1.00, 5.25) |
| Normal or overweight |  | 1.00 | 1.00 |  | 1.00 | 1.00 |  | 1.00 | 1.00 |
| Obese |  | 1.08 (0.88, 1.32) | 0.89 (0.75, 1.05) |  | 0.99 (0.61, 1.59) | 0.88 (0.66, 1.16) |  | 1.12 (0.53, 2.35) | 0.54 (0.29, 1.01) |
| Smoking |  |  |  |  |  |  |  |  |  |
| Never smoked |  | 1.00 | 1.00 |  | 1.00 | 1.00 |  | 1.00 | 1.00 |
| Former smoker |  | 1.11 (0.94, 1.32) | 1.00 (0.88, 1.13) |  | 0.75 (0.44, 1.27) | 1.09 (0.80, 1.48) |  | 0.77 (0.36, 1.62) | 1.85 (1.13, 3.05) |
| Active smoker |  | 1.33 (1.02, 1.74) | 1.40 (1.10, 1.78) |  | 1.66 (0.88, 3.12) | 1.58 (1.03, 2.43) |  | 0.64 (0.27, 1.52) | 1.46 (0.58, 3.67) |
| Alcohol Intake |  |  |  |  |  |  |  |  |  |
| Low or moderate |  | 1.00 | 1.00 |  | 1.00 | 1.00 |  | 1.00 | 1.00 |
| Heavy |  | 1.15 (0.79, 1.68) | 0.90 (0.65, 1.27) |  | 1.29 (0.62, 2.68) | 1.64 (0.39, 6.78) |  | 1.29 (0.36, 4.64) | 1.19 (0.49, 2.90) |
| Lonely |  | 1.09 (0.86, 1.37) | 1.03 (0.91, 1.17) |  | 0.93 (0.64, 1.35) | 1.18 (0.88, 1.58) |  | 1.43 (0.75, 2.73) | 0.82 (0.49, 1.39) |

Table A2 (Continued). Hazard ratios obtained from multistate model for mortality among decedents with observed dementia over the study period by race/ethnicity and gender.

|  |  | NH White | |  | NH Black | |  | Hispanic | |
| --- | --- | --- | --- | --- | --- | --- | --- | --- | --- |
|  |  | Men | Women |  | Men | Women |  | Men | Women |
| Characteristic |  |  |  |  |  |  |  |  |  |
| *Medical Conditions* |  |  |  |  |  |  |  |  |  |
| Diabetes |  | 1.21 (1.01, 1.46) | 1.20 (1.01, 1.44) |  | 1.72 (1.09, 2.73) | 1.52 (1.16, 1.98) |  | 2.56 (1.38, 4.73) | 2.88 (1.58, 5.26) |
| Hypertension |  | 1.18 (1.01, 1.37) | 1.09 (0.97, 1.23) |  | 1.16 (0.76, 1.75) | 1.29 (0.95, 1.73) |  | 1.13 (0.65, 1.98) | 1.15 (0.71, 1.86) |
| Stroke |  | 1.18 (0.94, 1.48) | 1.04 (0.88, 1.24) |  | 1.63 (1.03, 2.56) | 1.41 (0.95, 2.09) |  | 1.85 (0.87, 3.94) | 1.13 (0.36, 3.57) |
| Heart condition |  | 1.15 (0.99, 1.35) | 1.28 (1.12, 1.45) |  | 1.43 (0.85, 2.41) | 0.83 (0.61, 1.12) |  | 1.71 (0.75, 3.86) | 1.91 (1.01, 3.62) |
| Poor hearing |  | 0.99 (0.85, 1.16) | 1.03 (0.90, 1.17) |  | 0.73 (0.50, 1.06) | 0.97 (0.68, 1.38) |  | 1.37 (0.80, 2.32) | 1.28 (0.77, 2.12) |
| *Healthcare Utilization* |  |  |  |  |  |  |  |  |  |
| Any insurance |  | 0.88 (0.52, 1.46) | 1.16 (0.76, 1.77) |  | 1.01 (0.45, 2.25) | 1.60 (0.67, 3.82) |  | 0.79 (0.24, 2.67) | 1.05 (0.47, 2.33) |
| Doctor or hospital visit over past two years |  | 0.72 (0.48, 1.09) | 1.32 (1.00, 1.74) |  | 0.79 (0.41, 1.54) | 0.69 (0.37, 1.29) |  | 0.93 (0.22, 4.02) | 0.35 (0.13, 0.92) |

Table A3. Percentage of deaths attributable to risk factors among decedents without observed dementia over the study period by race/ethnicity and gender.

|  |  | NH White | |  | NH Black | |  | Hispanic | |
| --- | --- | --- | --- | --- | --- | --- | --- | --- | --- |
|  |  | Men | Women |  | Men | Women |  | Men | Women |
| Characteristic |  |  |  |  |  |  |  |  |  |
| *Socioeconomic Resources* |  |  |  |  |  |  |  |  |  |
| Educational Level |  |  |  |  |  |  |  |  |  |
| Less than high school or GED |  | 2.8 (-0.6, 6.2) | 5.0 (2.1, 7.7) |  | 11.9 (-13.8, 31.8) | 2.4 (-17.1, 18.7) |  | -68.7 (-196.3, 3.9) | -7.5 (-54.6, 25.2) |
| High school or above |  | Reference | Reference |  | Reference | Reference |  | Reference | Reference |
| Occupation |  |  |  |  |  |  |  |  |  |
| Never worked for pay |  | 1.8 (0.0, 3.6) | 1.8 (-1.2, 4.7) |  | 9.5 (-4, 21.3) | 10.2 (-8.5, 25.6) |  | 13.3 (-3.0, 27.1) | 11.1 (-27.2, 37.9) |
| Blue-collar |  | 5.0 (2.5, 7.4) | 0.4 (-1.8, 2.6) |  | 5.6 (-6.7, 16.5) | 4.9 (0.4, 9.2) |  | 10.5 (-8.2, 26.0) | -2.5 (-22.8, 14.4) |
| Lower white-collar |  | 3.5 (1.8, 5.2) | 3.6 (0.8, 6.3) |  | 7.4 (1.9, 12.5) | 1.5 (-3.9, 6.6) |  | 0.0 (-8.0, 7.5) | -3.3 (-16.5, 8.3) |
| Upper white-collar |  | Reference | Reference |  | Reference | Reference |  | Reference | Reference |
| Low neighborhood safety |  | -0.1 (-1.3, 1.1) | 0.3 (-0.8, 1.4) |  | 9.2 (2.5, 15.5) | 5.4 (-1.1, 11.5) |  | 9.5 (2.9, 15.7) | -9.2 (-26.0, 5.3) |
| Food Insecure |  | 0.5 (-0.2, 1.2) | 1.5 (0.8, 2.3) |  | 4.1 (-0.2, 8.3) | 9.2 (5.5, 12.8) |  | -2.8 (-9.3, 3.3) | 6.1 (-0.3, 12.1) |
| *Lifestyle Characteristics* |  |  |  |  |  |  |  |  |  |
| Physically inactive |  | 17.6 (13.9, 21.1) | 15.1 (10.0, 19.9) |  | 11.7 (-5.6, 26.2) | 20.2 (1.5, 35.3) |  | 2.8 (-32.9, 29) | 20.6 (-12.8, 44.1) |
| Body Mass Index |  |  |  |  |  |  |  |  |  |
| Underweight |  | 0.4 (0.2, 0.6) | 2.4 (1.8, 3.0) |  | 0.7 (-0.5, 1.9) | 2.9 (2.3, 3.6) |  | 0.1 (-0.1, 0.4) | 4.0 (2.4, 5.5) |
| Normal or overweight |  | Reference | Reference |  | Reference | Reference |  | Reference | Reference |
| Obese |  | 3.0 (0.7, 5.3) | 1.0 (-1.3, 3.3) |  | -4.7 (-15.7, 5.3) | 9.8 (-0.4, 19.0) |  | 11.8 (1.1, 21.3) | 7.1 (-8.6, 20.6) |
| Smoking |  |  |  |  |  |  |  |  |  |
| Never smoked |  | Reference | Reference |  | Reference | Reference |  | Reference | Reference |
| Former smoker |  | 16.7 (11.8, 21.3) | 10.0 (7.4, 12.4) |  | 11.2 (-2.7, 23.2) | 7.6 (-1.6, 16.0) |  | 12.4 (-8.9, 29.5) | 13.6 (1.2, 24.4) |
| Active smoker |  | 10.8 (9.7, 12.0) | 13.9 (13, 14.8) |  | 17.2 (11.6, 22.5) | 10.7 (6.3, 15.0) |  | 15.6 (9.1, 21.7) | 6.2 (3.2, 9.2) |
| Alcohol Intake |  |  |  |  |  |  |  |  |  |
| Low or moderate |  | Reference | Reference |  | Reference | Reference |  | Reference | Reference |
| Heavy |  | 1.7 (0.7, 2.8) | 0.4 (-0.6, 1.3) |  | -0.1 (-3.7, 3.3) | 2.1 (1.0, 3.3) |  | -2.1 (-13.1, 7.8) | 0.0 (-3.2, 3.1) |
| Lonely |  | 3.0 (1.2, 4.6) | 4.7 (2.8, 6.5) |  | -2.6 (-10.1, 4.5) | -0.1 (-7.3, 6.7) |  | 9.1 (2.2, 15.6) | -8.3 (-38.5, 15.3) |

Table A3 (Continued). Percentage of deaths attributable to risk factors among decedents without observed dementia over the study period by race/ethnicity and gender.

|  |  | NH White | |  | NH Black | |  | Hispanic | |
| --- | --- | --- | --- | --- | --- | --- | --- | --- | --- |
|  |  | Men | Women |  | Men | Women |  | Men | Women |
| Characteristic |  |  |  |  |  |  |  |  |  |
| *Medical Conditions* |  |  |  |  |  |  |  |  |  |
| Diabetes |  | 6.3 (5.0, 7.7) | 8.5 (7.4, 9.5) |  | 8.7 (2.3, 14.7) | 23.6 (19.1, 27.9) |  | 27.0 (22.9, 30.9) | 13.1 (8.5, 17.4) |
| Hypertension |  | 6.1 (2.1, 9.9) | 12.8 (9.2, 16.4) |  | 12.0 (-4.7, 26.0) | 13.6 (-5.3, 29.2) |  | -2.7 (-30.4, 19.2) | -21.3 (-52.3, 3.3) |
| Stroke |  | 3.5 (2.4, 4.5) | 3.7 (2.7, 4.7) |  | 4.7 (1.1, 8.2) | 5.0 (2.1, 7.8) |  | 3 (0.4, 5.6) | 1.3 (0.3, 2.2) |
| Heart condition |  | 11.7 (9.4, 14) | 9.6 (7.7, 11.5) |  | 16.0 (10.9, 20.8) | 15.6 (11.8, 19.2) |  | 5.7 (-3.0, 13.6) | 10.2 (4.9, 15.3) |
| Poor hearing |  | -3.4 (-6.7, -0.1) | 1.6 (-0.2, 3.4) |  | 0.4 (-6.5, 7.0) | -2.9 (-9.3, 3.2) |  | 5.5 (-6.6, 16.2) | 0.1 (-9.0, 8.5) |
| *Domain, Combined* |  |  |  |  |  |  |  |  |  |
| Socioeconomic Resources |  | 13.0 (9.4, 16.4) | 12.0 (6.8, 16.9) |  | 39.7 (26.8, 50.4) | 29.1 (14.2, 41.4) |  | -11.0 (-63.5, 24.7) | -3.3 (-70.5, 37.5) |
| Lifestyle Characteristics |  | 44.4 (39.5, 48.9) | 40.3 (35.9, 44.4) |  | 32.3 (8.4, 50.0) | 44.1 (27.8, 56.7) |  | 44.0 (15.3, 63.1) | 38.5 (-6.8, 64.6) |
| Medical Conditions |  | 21.9 (17.5, 26.0) | 30.2 (27.2, 33.1) |  | 34.2 (22.9, 43.8) | 44.1 (32.5, 53.8) |  | 34.8 (15.3, 49.8) | 8.8 (-13.2, 26.6) |
| **Total** |  | **62.1 (58.1, 65.7)** | **63.2 (59.7, 66.4)** |  | **72.3 (61.0, 80.4)** | **76.9 (67.4, 83.7)** |  | **60.3 (29.3, 77.7)** | **42.3 (-12.2, 70.4)** |

Table A4. Percentage of deaths attributable to risk factors among decedents with observed dementia over the study period by race/ethnicity and gender.

|  |  | NH White | |  | NH Black | |  | Hispanic | |
| --- | --- | --- | --- | --- | --- | --- | --- | --- | --- |
|  |  | Men | Women |  | Men | Women |  | Men | Women |
| Characteristic |  |  |  |  |  |  |  |  |  |
| *Socioeconomic Resources* |  |  |  |  |  |  |  |  |  |
| Educational Level |  |  |  |  |  |  |  |  |  |
| Less than high school or GED |  | -7.1 (-17.4, 2.3) | -1.3 (-6.7, 3.8) |  | -23.6 (-86.9, 18.2) | -26.3 (-64.2, 2.8) |  | -47.3 (-203.9, 28.6) | -6.0 (-129.6, 51.1) |
| High school or above |  | Reference | Reference |  | Reference | Reference |  | Reference | Reference |
| Occupation |  |  |  |  |  |  |  |  |  |
| Never worked for pay |  | -5.5 (-10.9, -0.4) | -7.3 (-14.4, -0.6) |  | -10.0 (-52.2, 20.5) | -7.0 (-32.4, 13.6) |  | 17.9 (-38.4, 51.2) | -99.9 (-486.6, 31.9) |
| Blue-collar |  | -10.9 (-21.0, -1.7) | -1.7 (-5.2, 1.7) |  | 1.9 (-32.3, 27.2) | -0.8 (-13.8, 10.8) |  | -10.1 (-75.2, 30.8) | -72.2 (-241.3, 13.1) |
| Lower white-collar |  | -3.8 (-7.9, 0.2) | -2.5 (-7.5, 2.4) |  | -3.7 (-18.2, 9.0) | -3.9 (-12.1, 3.8) |  | 11.0 (6.5, 15.4) | -2.1 (-15.3, 9.7) |
| Upper white-collar |  | Reference | Reference |  | Reference | Reference |  | Reference | Reference |
| Low neighborhood safety |  | 0.6 (-1.2, 2.4) | 0.0 (-1.7, 1.7) |  | -6.0 (-18.6, 5.2) | -0.3 (-7.9, 6.8) |  | -13.1 (-33.9, 4.5) | -11.3 (-28.9, 4.0) |
| Food Insecure |  | -0.4 (-2, 1.1) | 0.9 (-0.4, 2.3) |  | 2.5 (-4.9, 9.4) | 3.7 (-1.3, 8.5) |  | -6.2 (-22.5, 8) | 2.0 (-2.1, 5.9) |
| *Lifestyle Characteristics* |  |  |  |  |  |  |  |  |  |
| Physically inactive |  | 7.5 (0.2, 14.2) | 0.6 (-7.6, 8.1) |  | 9.1 (-14.5, 27.8) | 19.1 (2.0, 33.2) |  | 18.4 (-13.8, 41.4) | -43.4 (-129.6, 10.4) |
| Body Mass Index |  |  |  |  |  |  |  |  |  |
| Underweight |  | 0.2 (0, 0.4) | -0.4 (-1.7, 0.9) |  | 0.5 (0.5, 0.6) | 1.4 (-0.9, 3.7) |  | 0.0 (0.0, 0.0) | 2.8 (1.0, 4.6) |
| Normal or overweight |  | Reference | Reference |  | Reference | Reference |  | Reference | Reference |
| Obese |  | 1.2 (-2.2, 4.6) | -2.0 (-4.9, 0.9) |  | -0.2 (-11.1, 9.6) | -5.7 (-19.7, 6.6) |  | 1.9 (-10.6, 12.9) | -19.6 (-49.3, 4.2) |
| Smoking |  |  |  |  |  |  |  |  |  |
| Never smoked |  | Reference | Reference |  | Reference | Reference |  | Reference | Reference |
| Former smoker |  | 6.1 (-3.6, 14.9) | -0.1 (-4.1, 3.8) |  | -17.7 (-61.8, 14.4) | 2.8 (-7.6, 12.2) |  | -14.2 (-70.3, 23.4) | 13.9 (5.4, 21.6) |
| Active smoker |  | 3.7 (0.6, 6.6) | 2.8 (1.1, 4.5) |  | 13.1 (-0.5, 24.9) | 5.1 (1.2, 8.7) |  | -7.9 (-28.6, 9.5) | 2.0 (-2.1, 6.0) |
| Alcohol Intake |  |  |  |  |  |  |  |  |  |
| Low or moderate |  | Reference | Reference |  | Reference | Reference |  | Reference | Reference |
| Heavy |  | 0.8 (-1.2, 2.6) | -0.4 (-1.6, 0.9) |  | 3.0 (-4.9, 10.4) | 0.3 (-0.4, 1.1) |  | 1.2 (-4.3, 6.4) | 0.3 (-1.2, 1.8) |
| Lonely |  | 1.1 (-1.9, 4.0) | 0.8 (-2.5, 4) |  | -2.1 (-14.0, 8.5) | 4.6 (-3.2, 11.8) |  | 10.1 (-6.2, 24) | -9.1 (-39.4, 14.6) |

Table A4 (Continued). Percentage of deaths attributable to risk factors among decedents with observed dementia over the study period by race/ethnicity and gender.

|  |  | NH White | |  | NH Black | |  | Hispanic | |
| --- | --- | --- | --- | --- | --- | --- | --- | --- | --- |
|  |  | Men | Women |  | Men | Women |  | Men | Women |
| Characteristic |  |  |  |  |  |  |  |  |  |
| *Medical Conditions* |  |  |  |  |  |  |  |  |  |
| Diabetes |  | 3.4 (0.4, 6.3) | 2.3 (0.3, 4.4) |  | 9.3 (3.2, 15.0) | 10.3 (4.8, 15.4) |  | 29.7 (17.0, 40.5) | 24.5 (16.3, 32) |
| Hypertension |  | 7.0 (0.9, 12.7) | 4.5 (-1.7, 10.2) |  | 8.1 (-15.6, 26.9) | 15.6 (-2.4, 30.5) |  | 6.6 (-25.6, 30.6) | 7.6 (-20.2, 29.0) |
| Stroke |  | 1.9 (-0.5, 4.3) | 0.5 (-1.6, 2.6) |  | 5.8 (1.5, 10.0) | 2.7 (0.1, 5.3) |  | 10.2 (0.7, 18.7) | 1.1 (-8.9, 10.2) |
| Heart condition |  | 4.5 (-0.2, 9) | 5.7 (2.9, 8.3) |  | 6.3 (-1.7, 13.8) | -4.7 (-13.3, 3.3) |  | 12.8 (-3.3, 26.4) | 6.5 (1.8, 11.0) |
| Poor hearing |  | -0.3 (-6.4, 5.5) | 0.6 (-2.2, 3.4) |  | -9.1 (-22.4, 2.8) | -0.5 (-6.7, 5.3) |  | 9.4 (-5.2, 21.9) | 6.1 (-5.8, 16.7) |
| *Domain, Combined* |  |  |  |  |  |  |  |  |  |
| Socioeconomic Resources |  | -29.4 (-44.3, -16) | -11.9 (-24.5, -0.5) |  | -42.7 (-139.0, 14.8) | -35.8 (-79.8, -2.6) |  | -48.0 (-28.9.3, 43.7) | -209.8 (-846.3, -1.4) |
| Lifestyle Characteristics |  | 19.1 (6.9, 29.6) | 1.5 (-8.1, 10.2) |  | 5.4 (-48.7, 39.8) | 25.6 (7.8, 40.0) |  | 12.9 (-38.2, 45.1) | -51.6 (-160.1, 11.6) |
| Medical Conditions |  | 15.3 (7.1, 22.8) | 12.8 (6.7, 18.4) |  | 20.0 (-0.7, 36.4) | 22.4 (6.5, 35.6) |  | 49.6 (31.6, 62.9) | 39.6 (21.5, 53.5) |
| **Total** |  | 11.5 (-6.6, 26.5) | 4.0 (-11.1, 17.0) |  | -3.7 (-108.6, 48.4) | 22.7 (-8.9, 45.1) |  | 28.2 (-111.2, 75.6) | -1.6 (-7.0, 18.2) |

Table A5. Average annual number of incident dementia cases (95% confidence interval; 2000-2016) that may be theoretically preventable by eliminating risk factors by race/ethnicity and gender.

|  |  | NH White | |  | NH Black | |  | Hispanic | |
| --- | --- | --- | --- | --- | --- | --- | --- | --- | --- |
|  |  | Men | Women |  | Men | Women |  | Men | Women |
| Domain |  |  |  |  |  |  |  |  |  |
| Socioeconomic Resources |  | 62,496  (53,055-71,312) | 84,126  (62,738-10,3719) |  | 21,889  (17,213-25,439) | 33,654  (27,260-38,904) |  | 12,398  (8,381-14,682) | 25,862  (20,538-28,595) |
| Lifestyle Characteristics |  | 13,830  (-14,127-38,063) | 33,149  (6,733-57,320) |  | 12,670  (3,692-19,199) | 6,450  (-5,744-16,481) |  | -728  (-14,060-7,007) | 13,070  (5,718-18,324) |
| Medical Conditions |  | 27,088  (10,852-41,898) | 36,165  (18,403-52,871) |  | 5,960  (511-10,588) | 9,623  (697-17,270) |  | 4,806  (1,349-7,532) | 2,225  (-4,128-7,445) |
| Total |  | 88,886  (69,420-10,5354) | 13,0045  (10,4940-15,2186) |  | 28,827  (24,611-31,561) | 40,377  (32,671-46,125) |  | 13,847  (10,086-15,749) | 28,426  (25,093-30,020) |

Fig. A1 Percentage of incidence dementia cases attributable to risk factors and risk factor domains by race/ethnicity and gender.
